# Blood Flow Changes Coincide with Cellular Rearrangements during Blood Vessel Pruning in Zebrafish Embryos

**DOI:** 10.1371/journal.pone.0075060

**Published:** 2013-10-11

**Authors:** Eva Kochhan, Anna Lenard, Elin Ellertsdottir, Lukas Herwig, Markus Affolter, Heinz-Georg Belting, Arndt F. Siekmann

**Affiliations:** 1 Max Planck Institute for Molecular Biomedicine, Laboratory for Cardiovascular Patterning, Muenster, Germany; 2 Biozentrum der Universität Basel, Abteilung Zellbiologie, Basel, Switzerland; University of Queensland, Australia

## Abstract

After the initial formation of a highly branched vascular plexus, blood vessel pruning generates a hierarchically structured network with improved flow characteristics. We report here on the cellular events that occur during the pruning of a defined blood vessel in the eye of developing zebrafish embryos. Time-lapse imaging reveals that the connection of a new blood vessel sprout with a previously perfused multicellular endothelial tube leads to the formation of a branched, Y-shaped structure. Subsequently, endothelial cells in parts of the previously perfused branch rearrange from a multicellular into a unicellular tube, followed by blood vessel detachment. This process is accompanied by endothelial cell death. Finally, we show that differences in blood flow between neighboring vessels are important for the completion of the pruning process. Our data suggest that flow induced changes in tubular architecture ensure proper blood vessel pruning.

## Introduction

The vasculature is the first organ system to form during embryonic development and meets the challenge to grow and refine while it is already functioning. After the initial sprouting of blood vessels, remodeling ensures the formation of a more efficient vascular network [1]–[3]. While many of the key factors and genetic players regulating blood vessel sprouting are known, the mechanisms which control blood vessel remodeling and pruning are only poorly understood. The best-studied context is the regression of the hyaloid vasculature in mice [4]. Here, macrophages secrete a WNT ligand that induces apoptosis in endothelial cells, ultimately leading to the complete removal of this vascular structure [5], [6]. However, in many other contexts where vascular pruning has been described, such as the mouse retina [7], [8], the branchial arches [9] or the zebrafish brain [10], only a subset of blood vessel connections is removed. This raises the question: Which mechanism selects the blood vessels to be pruned? In addition, it is unclear how, once a given blood vessel is selected to disconnect and regress, the endothelial cells constituting this previously perfused vessel accomplish to seal it off from the active circulation without causing hemorrhage.

Here, we use time-lapse imaging to analyze the cellular mechanisms that take place during the pruning of a defined blood vessel in the eye of zebrafish embryos. Our analysis reveals that angiogenic sprouting initially leads to the formation of a Y-shaped blood vessel branch, which is subsequently resolved. This process entails the rearrangement of endothelial cells within the pruning blood vessel from a multicellular to a partially unicellular tube. We show that blood flow is an important regulator of the pruning event. Importantly, our results suggest that loss of perfusion in itself does not lead to blood vessel pruning, but that pruning might be facilitated by the establishment of differences in blood flow between vessels in a branch point.

## Results and Discussion

### Live Imaging of Blood Vessel Pruning

**Figure 1:**
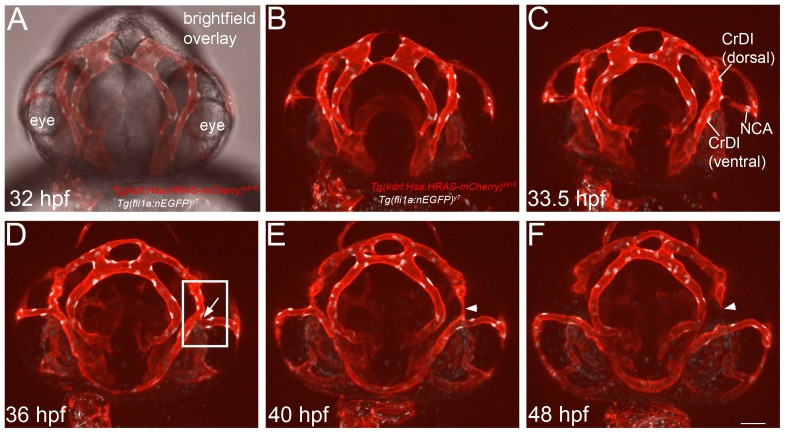
Pruning of the Cranial Division of the Internal Carotid Artery (CrDI) during eye blood vessel development in zebrafish embryos. Still images of a frontal view time-lapse movie of *Tg(kdrl:Hsa.HRAS-mcherry)^s916^; Tg(fli1a:nEGFP)^y7^* zebrafish embryos. (A) Overlay of brightfield image with fluorescent channels. (B) Fluorescent channel only. (C) Onset of the connection of the Nasal Ciliary Artery (NCA) to the CrDI at 33.5 hpf. (D) Connection of the NCA has completed (arrow). Box indicates area imaged in Figure 2. (E) Collapse of endothelial lumen in the dorsal CrDI (arrowhead) at 40 hpf. (F) Completion of CrDI pruning (arrowhead). Scale bar = 50 µm.

To better understand vascular remodeling, we set out to identify blood vessels that undergo pruning during zebrafish embryonic development. We performed time-lapse analysis of the eye vasculature between 32 hours post fertilization (hpf) and 48 hpf in *Tg(kdrl:Hsa.HsRAS-mcherry)^sa916^*; *Tg(fli1a:nEGFP)^y7^* fish to label endothelial cell membranes and nuclei (Figure 1). These movies revealed that a discrete portion of the Cranial Division of the Internal Carotid Artery (CrDI) was pruned in a consistent manner (Figure 1, see also Movie S1). Initially, the Nasal Ciliary Artery (NCA) connected to the CrDI, dividing it into a dorsal and a ventral part (Figure 1C, arrow in D, naming according to [11]). After branch point establishment, we observed a collapse of endothelial lumen in the dorsal CrDI (Figure 1E, arrowhead), followed by blood vessel regression (Figure 1E, F, arrowheads). Imaging several zebrafish larvae furthermore showed that this pruning event occurred in a highly reproducible manner, facilitating the investigation of the effects of experimental manipulations on the regression of this blood vessel.

### Blood Vessels Rearrange from Multicellular to Partially Unicellular Tubes Prior to Pruning

**Figure 2:**
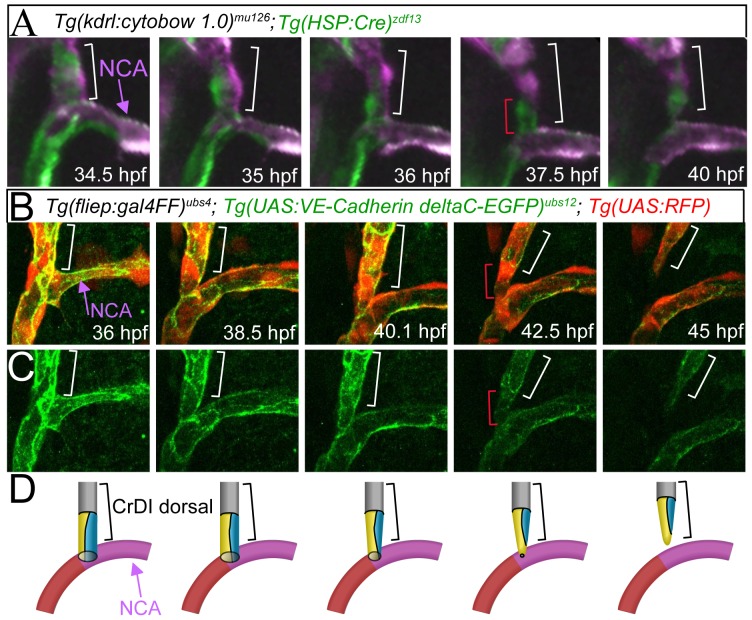
Endothelial cells rearrange from a multicellular into a partially unicellular tube during blood vessel pruning. (A) Still images from a time-lapse movie of *Tg(kdrl:cytobow1.0)^mu125^*; *Tg(HSP:Cre)^zdf13^* embryos. Imaged area indicated with box in Figure 1D. Bracket marks dorsal CrDI. At the 37.5 hpf time point, note unicellular connection between the dorsal CrDI and the NCA (single green cell, marked with red bracket). (B) Merged still images from time-lapse imaging of *Tg(UAS:RFP)*; *Tg(fliep:Gal4FF)^ubs4^*; *Tg(UAS:VE-cadherin-deltaC-EGFP)^ubs12^* between 36 and 45 hpf. Red bracket at 42.5 hpf indicates unicellular connection between dorsal CrDI and NCA. (C) Still images from *Tg(UAS:VE-cadherin-deltaC-EGFP)^ubs12^* expression corresponding to images in B. (D) Schematic drawing of cell rearrangements shown in B, C. Brackets label dorsal CrDI, purple arrow highlights NCA.

To better understand the cellular mechanisms that accompany blood vessel pruning, we generated *Tg(kdrl:cytobow1.0)^mu125^* fish (see Materials and Methods) to label individual endothelial cells in different colors. Initially, endothelial cells of the dorsal CrDI formed a multicellular tube, with two cells surrounding the vascular lumen (Figure 2A, 34.5 hpf time point, bracket indicates dorsal CrDI with a green and a purple cell, NCA with purple arrow, see also Movie S2). Subsequently, cell rearrangements led to the establishment of a partially unicellular tube in the dorsal CrDI, as evidenced by a single green cell making a connection to the NCA (Figure 2A, 37.5 hpf time point, red bracket). Afterwards, this cell started to disconnect from the NCA, thus causing the separation of the two vessels (Figure 2A, 40 hpf time point). These observations suggest that CrDI pruning occurs via an intermediate step, in which a segment of a multicellular tube changes its architecture into a unicellular tube.

To corroborate our findings, we used *Tg(UAS:VE-cadherin-deltaC-EGFP)^ubs12^* fish [12] to analyze endothelial cell junctional dynamics during CrDI pruning. Initially, all blood vessels within the Y-shaped branch consisted of multicellular tubes, as defined by the presence of two or more parallel cell junctions along the vessel axis (Figure 2B, C, 36 hpf to 40.1 hpf time points; see Figure 2D for schematized view, Movie S3). At the onset of dorsal CrDI pruning, one of the two cells at the contact point retracted into the dorsal CrDI, thus transforming the initial multicellular tube into a partially unicellular or seamless tube, as evidenced by the absence of a junction (red brackets in Figures 2B, C, 42.5 hpf time point, Movie S3). Finally, the junctional ring at the attachment site constricted into a single point as the CrDI detached (Figure 2B, C, 45 hpf time point). Thus, as evidenced by our single cell labeling experiments and by the analysis of junctional dynamics, CrDI pruning consists of a multi-step process that involves the rearrangement of endothelial cells from a multicellular into a partially unicellular tube prior to blood vessel detachment and regression.

### Macrophage Independent Endothelial Cell Apoptosis Contributes to Blood Vessel Pruning

**Figure 3:**
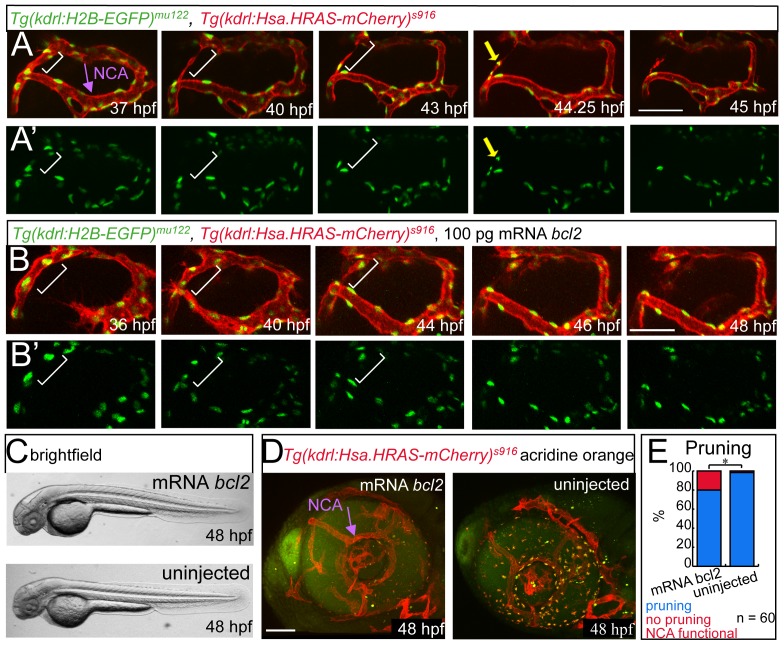
Endothelial cell apoptosis during CrDI pruning. All images in lateral view with anterior to the left. (A) Stills of time-lapse imaging of *Tg(kdrl:H2B-EGFP)^mu122^*, *Tg(kdrl:Hsa.HRAS-mCherry)^s916^* from 37–45 hpf. (A’) *Tg(kdrl:H2B-EGFP)^mu122^* channel only. White brackets indicate dorsal CrDI. Yellow arrow in (A) and (A’) at 42.25 hpf time point points to dying cell. (B) Stills of time- lapse imaging of *Tg(kdrl:H2B-EGFP)^mu122^*; *Tg(kdrl:Hsa.HRAS-mCherry)^s916^* embryos from 36–48 hpf injected with *bcl2* RNA. No dying cells are detectable in the CrDI. (B’) *Tg(kdrl:H2B-EGFP)^mu122^* channel only. (C) Brightfield images of *bcl2* mRNA or uninjected embryos. (D) Acridine orange staining on *bcl2* mRNA injected or control embryos. (E) Quantification of CrDI pruning at 48 hpf.

Previous studies had shown that macrophage induced apoptosis was essential for the regression of hyaloid vessels during mouse development [5], [13]–[16]. We therefore analyzed if apoptosis was similarly important during CrDI pruning. Observation of endothelial cell nuclei in 9 different time-lapse movies revealed that each regressing dorsal CrDI contained 3–4 endothelial cell nuclei (counted in 9 time lapse movies, Table S1 in File Supplementary Tables). Of these, 1–2 endothelial cells died during CrDI regression (Figure 3A, A’, cell undergoing apoptosis is labeled by yellow arrow at time point 44.25 hpf, which marks nuclear fragmentation, Movie S4). The remaining CrDI cells migrated towards the dorsally located Primordial Midbrain Channel and in 2 out of 9 movies into the ventral CrDI. Therefore, CrDI pruning is accompanied by endothelial cell death and incorporation of the remaining endothelial cells into neighboring blood vessels.

To address the question of whether the observed apoptosis was required for blood vessel regression, we overexpressed the apoptosis inhibitor *bcl2* [17]. This blocked endothelial cell apoptosis (6 out of 6 time-lapse movies; Figure 3B, B’) without affecting overall morphology (Figure 3C). In addition, the number of apoptotic cells in all tissues was reduced, as shown by Acridine Orange staining (Figure 3D). Blocking apoptosis led to a pruning defect in 20% of the embryos analyzed (Figure 3E, Table S2 in File S1). However, we observed that in the *bcl2* injected embryos more endothelial cells migrated into the neighboring blood vessels. We therefore conclude that even though endothelial cell apoptosis occurs during CrDI pruning, it is not absolutely required for the completion of this process.

Next, we analyzed if the observed endothelial cell apoptosis is dependent on macrophages. We injected morpholinos (MO) targeting the macrophage specific transcription factor pu1.1 [18]. *pu.1* MO injected embryos had normal regression in 95.1% of 352 eyes analyzed, compared to 97.8% in control embryos (Table S3 in File S1). These results indicate that macrophages do not influence CrDI regression. This suggests that the mechanisms controlling CrDI pruning are distinct from those regulating the regression of the hyaloid vasculature.

### Changes in Hemodynamics Regulate Blood Vessel Pruning

**Figure 4:**
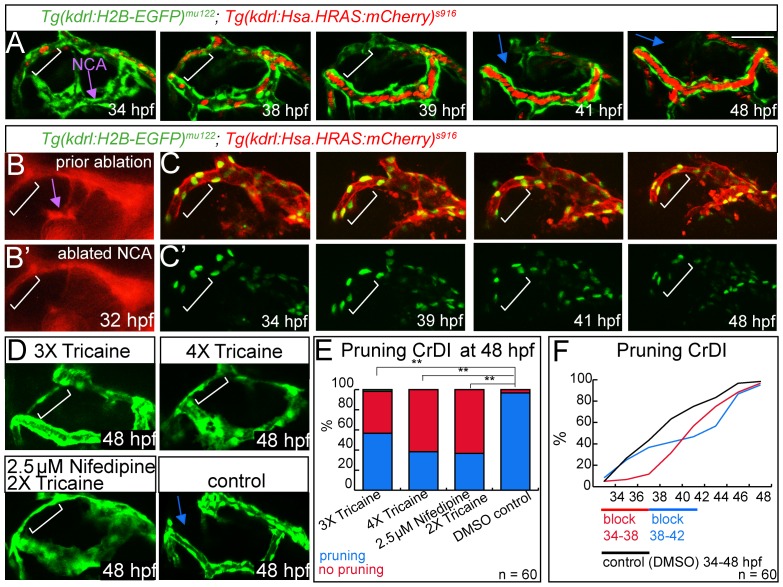
Pruning of the CrDI is flow dependent. Lateral views, anterior to the left, dorsal to the top. (A) Still images of time-lapse of *Tg(kdrl:EGFP)^s843^*; *Tg(gata1a:DsRed)^sd2^* from 34–48 hpf. White brackets indicate dorsal CrDI, purple arrow indicates NCA. Note reduction of blood flow in dorsal CrDI (39 hpf time point) prior to regression (blue arrows at 41 and 48 hpf time points). (B) Laser ablation of NCA at 32 hpf (purple arrow), compare to (B’). (C) Time-lapse imaging from 34–48 hpf of *Tg(kdrl:H2B-EGFP)^mu122^*; *Tg(kdrl:Hsa.HRAS-mCherry)^s916^* after NCA ablation and *Tg(kdrl:H2B-EGFP)^mu122^* only (C’). Note persistent CrDI (white brackets). (D) Blocking of heartbeat by indicated drug treatments from 30–48 hpf and imaging at 48 hpf. (E) Quantification of drug treatments shown in D. (F) Influence of 4 hr block of heartbeat followed by drug wash out on CrDI regression at indicated time points. Scale bar = 50 µm.

Previous studies analyzing blood vessel pruning in zebrafish brains, mice retinae and airways showed that loss of blood vessel perfusion precedes blood vessel regression [7], [10], [19], [20]. We therefore monitored changes in blood flow patterns between 36 and 48 hpf in CrDI and NCA blood vessels (Figure 4A, Movie S5). Prior to connection of the NCA to the CrDI, no erythrocytes were detected within the sprouting NCA (Figure 4A, 34 hpf time point). Upon connection to the CrDI, red blood cells entered the NCA, while blood flow through the dorsal CrDI became progressively weaker (Figure 4A, 38 hpf). Eventually, blood flow was almost completely rerouted through the NCA, while the dorsal CrDI was only minimally perfused (Figure 4A, 39 hpf time point). Subsequently, the dorsal CrDI regressed (Figure 4A, 41 and 48 hpf, labeled with blue arrow). Therefore, connection of the NCA to the CrDI leads to distinct changes in blood flow within the dorsal CrDI and the NCA, respectively. Furthermore, a reduction of blood flow preceded pruning of the dorsal CrDI.

Based on these observations, we set out to determine whether manipulating blood flow patterns would influence CrDI pruning. We asked, whether we could establish forced blood flow through the dorsal CrDI by laser ablating the NCA. We performed the laser ablation on one side of experimental embryos, while we left the other side as a control. We observed a persistent dorsal CrDI, which continued to carry blood flow (data not shown), on the side on which we had ablated the NCA, but not on the control side (Figure 4B, B’, 4C, C’, Movie S6). These results suggest that in the absence of an NCA connection, forced blood flow through the dorsal CrDI prevents blood vessel pruning.

We next asked whether inhibition of blood flow would lead to precocious pruning of the CrDI. We manipulated blood flow by altering the heartbeat either by drug treatment or by injecting zebrafish embryos with MO targeting *cardiac troponin t2a* (*tnnt2a*), which never establish a heartbeat [21]. Surprisingly, in *tnnt2a* MO injected embryos, the CrDI failed to prune in about 50% of cases (Table S4 in File Supplementary Tables). In embryos that were either treated with 4x Tricaine or with Nifedipine to stop the heartbeat [22], the CrDI similarly failed to prune in 60% of cases (Figure 4D, white brackets label persistent CrDI, blue arrow marks regressed CrDI in control embryos, Figure 4E, Table S5 in File Supplementary Tables). Reducing the heartbeat by 20% through treatment with 3x Tricaine blocked CrDI regression in around 40% of the embryos (Figure 4E, Tables S5, S6 in File S1). To test, whether this block in CrDI pruning was reversible, we performed wash out experiments, in which we blocked the heartbeat for 4 hours from 34 to 38 hpf and from 38 to 42 hpf. For both treatment regimes, CrDI regression was blocked during the time without heartbeat, but started after drug wash out and reached almost normal levels at 48 hpf (Figure 4F, Table S7 in File S1). These findings indicate that solely losing perfusion in a given blood vessel is not sufficient to trigger pruning in a situation of global block of blood flow, in which adjacent blood vessels would also not be perfused.

Our study is the first to describe the cellular rearrangements occurring during blood vessel pruning and establishes a sequence of events during which a multicellular tube is transformed into a partially unicellular tube prior to blood vessel detachment. This change in blood vessel architecture might be necessary in order to ensure proper blood vessel pruning without vessel rupture or hemorrhage. A recent report describes that during blood vessel fusion, endothelial cells rearrange from a unicellular to a multicellular tube [12]. Therefore, the changes in blood vessel architecture that occur during blood vessel pruning appear to be the same as for blood vessel fusion, but in the reverse order.

Previous studies showed that pruning of early forming hindbrain vessels in zebrafish embryos occurs normally in the absence blood flow [23], [24], while at later stages, loss of perfusion leads to accelerated blood vessel pruning [10]. We observed loss of blood flow prior to CrDI pruning, while forcing blood flow through the CrDI led to a persistent CrDI. Of interest, global block of blood flow did not lead to precocious CrDI pruning, but to an impairment of CrDI pruning. These results suggest that it is not loss of blood flow *per se* that determines if a given blood vessel will prune. Rather, the difference in blood flow between neighboring blood vessels might be sensed by endothelial cells, ultimately triggering the pruning of the less perfused vessel. Our blood flow blocking experiments furthermore suggest that blood flow directly acts on endothelial cells during blood vessel pruning, since pruning rapidly resumed after reestablishing the heartbeat. We note that in embryos with a global block in blood flow, CrDI pruning still occurred in about 40% of analyzed embryos, suggesting the existence of flow independent mechanisms controlling blood vessel pruning. The identification of these mechanisms and their potential integration with flow-based processes will greatly advance our understanding of blood vessel pruning and vascular remodeling.

## Materials and Methods

### Zebrafish Strains

Zebrafish were maintained as described previously [25]. All animal experiments were performed in accordance with the relevant laws and institutional guidelines at the Max Planck Institute for Molecular Biomedicine. All protocols concerning animal maintenance and handling were approved by the state of North Rhine-Westphalia (Germany), and all efforts were made to minimize suffering. Embryos were staged by hours post-fertilization (hpf) at 28.5°C [26]. The following transgenic lines were used: *Tg(kdrl:EGFP)^s843^* [27], *Tg(kdrl:Hsa.HRAS-mCherry)^s916^* [28], *Tg(fli1a:nEGFP)^y7^* [29], *Tg(gata1a:DsRed)^sd2^* [30], *Tg(UAS:RFP)* (kindly provided by Kawakami lab), *Tg(HSP:Cre)^zdf13^* [31]; *Tg(UAS:VE-cadherin-deltaC-EGFP)^ubs12^* [12], *Tg(kdrl:cytobow1.0)^mu126^* and *Tg(kdrl:H2B-EGFP)^mu122^* (this study).

### Generation of *Tg(kdrl:H2B-EGFP)^mu122^* and *Tg(kdrl:cytobow1.0)^mu126^* Zebrafish

pCS2-H2B-EGFP [32] containing *Danio rerio* Histone2B (H2B) fused in frame to EGFP was used to PCR amplify and subclone H2B-EGFP into pBluescript. Thereafter, H2B-EGFP was cloned via EcoRV and EcoRI into pKdrl:MCS-Tol2 [33] to generate pKdrl:MCS-Tol2-H2B-EGFP. DNA was isolated by Maxiprep, purified with PCR purification kit (Qiagen) and AB embryos at the one cell stage were injected with 30 ng DNA pKdrl:MCS-Tol2-H2B-EGFP and 50 ng mRNA *tol2* transposase (Message Machine kit, Ambion) [34]. At 24 hpf, embryos were screened for vascular-specific EGFP expression. Adult founders with germline transmission were identified by outcrosses and the brightest founder was maintained to establish *Tg(kdrl:H2B-EGFP)^mu122^*.

In order to generate *Tg(kdrl:cytobow1.0)^mu126^* fish, we used NheI and DraIII to release the cassette containing the fluorescent proteins from CMV Brainbow 1.0 “L” (Addgene 18721) [35]. Subsequently, we filled in the restriction sites with T4 DNA polymerase and ligated the blunted insert into EcoRV digested pkdrl:MCS-Tol2 plasmid [33]. We screened for adult founders with germline transmission by analyzing vascular tdTomato expression at 36 hpf and maintained the brightest founder to establish *Tg(kdrl:cytobow1.0)^mu126^*. Cytobow 1.0L constructs contain two floxed fluorescent proteins (tdTtomato and Cerulean) and Yellow Fluorescent Protein. Cre-mediated recombination between individual Lox-P sites can lead to the expression of different combinations of fluorescent proteins, as previously shown [35]. In order to gain temporal control over Cre activity, we crossed *Tg(kdrl:cytobow1.0)^mu125^* fish to *Tg(HSP:Cre)^zdf13^* fish. This resulted in 25% double transgenic offspring. Embryos were subsequently heat shocked at 90% epiboly and those with differently labeled endothelial cells in anterior blood vessels were chosen for time-lapse analysis.

### Analysis of Regression of CrDI Artery and Statistics

We used *Tg(kdrl:EGFP)^s843^* embryos to analyze the regression of the dorsal CrDI. Regression was analyzed at 48 hpf under a stereomicroscope or via confocal microscopy. Only embryos with normal blood flow patterns at 36 hpf were included in the analysis. We defined regression as complete loss of endothelial connection between CrDI and PMBC. Each experiment was performed in triplicates with 20 eyes being analyzed per experiment. Quantification charts display the average values of three experiments. Data were evaluated with two-tailed unpaired Student’s t test and significance was calculated for regression.

### Time-lapse, Confocal Microscopy, and Vessel Ablation

Live imaging of endothelial cell junctions using the *Tg(UAS:VE-cadherin-deltaC-EGFP)^ubs12^* line was performed as previously described [12]. For other *in vivo* imaging, live embryos were mounted essentially as described previously [36]. Briefly, embryos were mounted in 1% low melting point agarose in E3 embryo medium with 168 mg/l Tricaine (1X) for anesthetization and 0.003% phenylthiourea to inhibit pigmentation. Imaging was carried out at an SP5 confocal microscope using a 20x objective (Leica Microsystems). We used a heated microscope chamber at 28.5°C for recording time- lapse movies. Stacks were taken every 15 min with a step size of 1.5–2.5 µm. Confocal stacks and time-lapse movies were analyzed using Imaris Software (Bitplane), ImageJ (NIH), and Quicktime Player Pro. Vessels in close proximity to the CrDI were cropped away to increase clarity. Ablation of the NCA artery was carried out between 31–33 hpf using a Zeiss epifluorescence microscope equipped with a PALM micro laser dissection system. The laser beam was applied until fluorescence of *Tg(kdrl:H2B-EGFP)^mu122^* disappeared in the ablated vessel. After NCA ablation, we monitored the embryos for PMBC integrity and normal blood flow.

### Morpholinos (MOs) and mRNA Injection

MO were obtained from Gene-Tools and dissolved in dH_2_O. Embryos were injected at one cell stage with 4 ng MO *tnnt2a* (silent heart 5′ CATGTTTGCTCTGATCTGACACGCA 3′) [21] or 4 ng MO *pu.1* (5′-GATATACTGATACTCCATTGGTGGT-3′) [18]. As a control, we injected standard control morpholinos (5′-CCTCTTACCTCAGTTACAATTTATA-3′). *Danio rerio bcl2* mRNA was transcribed from pCS2-Bcl2 (kindly provided by J. Hillmer) using Message Machine kit (Ambion) and injected into one cell stage with a final amount of 100 pg.

### Acridine Orange (AO) Staining and Drug Treatments

For AO staining, embryos were incubated for 30 min in 10 µg/ml AO in E3 at 28.5°C, followed by washing for 30 min. Drug treatments were carried out on dechorionated embryos in E3 medium. To completely stop the heart at various time points, we either treated zebrafish embryos with 4X Tricaine or with 2X Tricaine in addition to 2.5 µM Nifedipine [22]. To slow down heartbeat, we treated embryos with 3X Tricaine. All drug treatments were performed from 30 to 48 hpf, prior to the onset of CrDI regression. For wash out experiments, drugs were removed and embryos were rinsed twice with E3 medium. We observed that after drug wash out, blood flow was re-established within 1 hour.

## Supporting Information

File S1 **Supporting tables.**
(DOC)

Movie S1 **(Related to** **Figure 1** **): Time lapse imaging of early eye blood vessel development.** Time-lapse imaging of *Tg(kdrl:Hsa.HRAS-mCherry)^s916^*; *Tg(fli1a:nEGFP)^y7^* embryos. Images were taken from 32–48 hpf at 15 minute time intervals. Endothelial cell membranes are marked by red fluorescence, while endothelial cell nuclei are marked in white. Arrows indicate the forming Nasal Ciliary Artery (NCA). After connection of the NCA (indicated by arrows) to the Cranial Division of the internal carotid artery (CrDI), the dorsal CrDI prunes (arrowheads).
(MOV)

Movie S2 **Cellular rearrangements during CrDI pruning based on labeling of individual endothelial cells (Related to** **Figure 2A** **).** Time-lapse imaging of *Tg(kdrl:cytobow 1.0)^mu126^*; *Tg(HSP:Cr)^zdf13^* embryos. Images were taken from 34.5–40 hpf at 15 minute time intervals. Distinct endothelial cells were false colour labeled in green and magenta, respectively. Note absence of magenta cell in dorsal CrDI during unicellular configuration prior to CrDI pruning.
(MOV)

Movie S3 **Cellular rearrangements during CrDI pruning based on labeling of endothelial cell junctions (Related to** **Figure 2B, C** **).** Time-lapse imaging with *Tg(UAS:RFP); Tg(fliep:Gal4FF)^ubs4^*; *Tg(UAS:VE-cadherin-deltaC-EGFP)^ubs12^* in a frontal-lateral orientation between 36 and 45 hpf. In the overlay channel, endothelial cells are labeled in red and endothelial junctions are shown in green. Middle panels show junctional labeling alone with individual cells and junctions colour coded to illustrate junctional dynamics during CrDI pruning. Right panel shows RFP channel alone, marking endothelial cell bodies.
(MOV)

Movie S4 (MOV)

Movie S5 (MOV)

Movie S6 **Laser ablation of the NCA and influence on CrDI pruning (Related to** **Figure 4B** **).** Laser ablation of the NCA was carried out at 32 hpf and time-lapse imaging was performed from 34–48 hpf in *Tg(kdrl:H2B-EGFP)^mu122^*, *Tg(kdrl:Hsa.HRAS-mCherry)^s916^* double transgenic zebrafish, labeling all endothelial nuclei in green and endothelial membranes in red. In the absence of a functional NCA, the CrDI fails to prune.
(MOV)
