# Correction: Blood Flow Changes Coincide with Cellular Rearrangements during Blood Vessel Pruning in Zebrafish Embryos

**DOI:** 10.1371/annotation/9e16dd10-9d37-4d4f-85b2-0811852b15f0

**Published:** 2014-01-30

**Authors:** Eva Kochhan, Anna Lenard, Elin Ellertsdottir, Lukas Herwig, Markus Affolter, Heinz-Georg Belting, Arndt F. Siekmann

The title and descriptions are missing for Movie S4 and Movie S5. Please find the missing information below. 

**Movie S4: Cell death and migration during CrDI pruning (Related to Figure 3A).**

Time-lapse imaging of *Tg(kdrl:H2B-EGFP)^mu122^* labeling all endothelial nuclei in green and *Tg(kdrl:Hsa.HRAS-mCherry)^s916^* labeling all endothelial membranes in red from 37-45 hpf. Time interval between frames is 15 minutes. Endothelial cell nucleus undergoing apoptosis is labeled with arrowhead; cell fragments with small arrowheads. Arrow marks fast moving Cherry positive cell (potentially phagocytic), which briefly moves into the region, where cell death occurs.

**Movie S5: Blood flow during CrDI pruning (Related to Figure 4A).**

Stills of time-lapse imaging of *Tg(kdrl:EGFP)^s843^* labeling all vessels in green and *Tg(gata1a:DsRed)^sd2^* labeling erythrocytes in red from 34-48 hpf. Note initiation of blood flow in the NCA, while blood flow in the CrDI gets weaker shortly after.

