# Supplementary material for: Blood Flow Changes Coincide with Cellular Rearrangements during Blood Vessel Pruning in Zebrafish Embryos
Source: PLoS One. 2013 Oct 11;8(10):e75060. doi: 10.1371/journal.pone.0075060 (PMC3795766; doi:10.1371/journal.pone.0075060)
Supplement: File S1 — Supporting tables. (DOC) [file pone.0075060.s001.doc]

Table S1: Counting of cells in dorsal CrDI at 36 hpf and analysis of cell fate (apoptosis or migration) during pruning of dorsal CrDI up to 48 hpf. stdev = standard deviation.

|  | **36 hpf** | **36-48 hpf** |  | |  |  |
| --- | --- | --- | --- | --- | --- | --- |
|  | **CrDI dorsal** | **apoptosis** | **migration to PMBC** | **migration ventral CrDI** | | **proliferation** |
|  | 4 | 2 | 3 | 0 | | 1 |
|  | 3 | 1 | 2 | 0 | | 0 |
|  | 4 | 1 | 3 | 0 | | 0 |
|  | 4 | 2 | 1 | 1 | | 0 |
|  | 4 | 1 | 3 | 0 | | 0 |
|  | 3 | 1 | 1 | 1 | | 0 |
|  | 2 | 2 | 1 | 0 | | 1 |
|  | 4 | 1 | 3 | 0 | | 0 |
|  | 3 | 2 | 1 | 0 | | 0 |
| **average** | 3.4 | 1.4 | 2.0 | 0.2 | | 0.2 |
| **stdev** | 0.7 | 0.5 | 1.0 | 0.4 | | 0.4 |

Table S2 (for Figure 3E): Scored pruning of CrDI upon Bcl2 overexpression at 48 hpf. Numbers display counted values of 3 independent experiments and average in percent.

|  | **regression** | **no regression NCA OK** | **no regression NCA -** |  |
| --- | --- | --- | --- | --- |
| **Bcl2** | 15 | 5 | 0 |  |
|  | 16 | 4 | 0 |  |
|  | 17 | 3 | 0 |  |
| **uninjected** | 19 | 1 | 0 |  |
|  | 20 | 0 | 0 |  |
|  | 20 | 0 | 0 |  |
| **average in %** |  |  |  | **p-value** |
| **Bcl2 in %** | 80.0 | 20.0 | 0.0 | 0.005328 |
| **uninjected in %** | 98.3 | 1.7 | 0.0 |  |

Table S3: Scored pruning of CrDI in embryos lacking macrophages at 48 hpf. Numbers display counted values of 3 independent experiments and average in percent.

|  | **regression** | **no regression NCA OK** | **no regression NCA -** |  |
| --- | --- | --- | --- | --- |
| **MO pu.1** | 62 | 8 | 2 |  |
|  | 168 | 0 | 0 |  |
|  | 111 | 0 | 1 |  |
| **MO control** | 49 | 0 | 1 |  |
|  | 136 | 2 | 2 |  |
|  | 173 | 0 | 3 |  |
| **average in %** |  |  |  | **p-value** |
| **MO pu.1 in %** | 95.1 | 3.7 | 1.2 | 0.5756 |
| **MO control in %** | 97.8 | 0.5 | 1.7 |  |

Table S4: Scoring of pruning of CrDI in embryos without blood flow at 48 hpf in three individual experiments. Average value of 3 experiments is given in percent.

|  | **regression** | **no regression NCA OK** | **no regression NCA -** |  |
| --- | --- | --- | --- | --- |
| **untreated NCA, MO tnnt2** | 7 | 7 | 2 |  |
|  | 7 | 10 | 2 |  |
|  | 8 | 9 | 3 |  |
| **untreated NCA, MO control** | 20 | 0 | 0 |  |
|  | 19 | 1 | 0 |  |
|  | 19 | 1 | 0 |  |
| **average in %** |  |  |  | **p-value** |
| **MO tnnt2 in %** | 40.0 | 47.3 | 12.7 | 0.000014 |
| **MO control in %** | 96.7 | 3.3 | 0.0 |  |

Table S5 (for Figure 4E): Scored pruning of CrDI after block of blood flow at 48 hpf. Numbers display counted values of 3 independent experiments and average in percent.

|  | **regression** | **no regression NCA OK** | **no regression NCA -** |  | |
| --- | --- | --- | --- | --- | --- |
| **3X Tricaine** | 11 | 9 | 0 |  | |
|  | 12 | 8 | 0 |  | |
|  | 11 | 8 | 1 |  | |
| **4X Tricaine** | 8 | 12 | 0 |  | |
|  | 8 | 12 | 0 |  | |
|  | 7 | 13 | 0 |  | |
| **Nifedipine + 2X Tricaine** | 8 | 12 | 0 |  | |
|  | 6 | 14 | 0 |  | |
|  | 8 | 12 | 0 |  | |
| **control** | 19 | 1 | 0 |  | |
|  | 20 | 0 | 0 |  | |
|  | 19 | 1 | 0 |  | |
| **average in %** |  |  |  | **p-value** |  |
| **3X Tricaine in %** | 56.7 | 41.7 | 1.7 | 0.00007 |  |
| **4X Tricaine in %** | 38.3 | 61.7 | 0.0 | 0.00002 |  |
| **Nifedipine + 2X Tricaine in %** | 36.7 | 63.3 | 0.0 | 0.00009 |  |
| **control in %** | 96.7 | 3.3 | 0.0 |  |  |

Table S6: Counting of heartbeat per minute at 48 hpf after 3x Tricaine treatment 30-48 hpf. Numbers display counted values of 4 independent experiments and average.

| **heartbeat /min at 48 hpf** | **clutch 1** | **clutch 2** | **clutch 3** | **clutch 4** | |
| --- | --- | --- | --- | --- | --- |
|  |  |  |  |  | |
| **control** | 96 | 100 | 108 | 120 | |
|  | 120 | 112 | 120 | 112 | |
|  | 120 | 120 | 116 | 108 | |
|  | 116 | 120 | 96 | 116 | |
|  | 120 | 108 | 104 | 100 | |
|  | 104 | 116 | 116 | 112 | |
|  | 100 | 112 | 108 | 116 | |
|  | 120 | 116 | 116 | 104 | |
|  | 120 | 112 | 112 | 112 | |
|  | 100 | 112 | 120 | 108 | |
|  |  |  |  |  | |
| **3X Tricaine** | 96 | 96 | 88 | 100 | |
|  | 92 | 84 | 92 | 92 | |
|  | 92 | 100 | 96 | 96 | |
|  | 88 | 96 | 76 | 88 | |
|  | 96 | 88 | 92 | 76 | |
|  | 88 | 92 | 96 | 84 | |
|  | 84 | 88 | 76 | 96 | |
|  | 96 | 88 | 80 | 88 | |
|  | 88 | 84 | 96 | 92 | |
|  | 92 | 92 | 80 | 84 | |
|  | **average** | **p-value** |  | |  |
| **control** | 112 |  |  | |  |
| **3X Tricaine** | 90 | 2.08 E-23 |  | |  |
|  |  |  |  | |  |
|  |  |  |  | |  |
|  |  |  |  | |  |

Table S7 (for Figure 4F): Scoring of CrDI pruning from 34-48 hpf in three individual experiments (clutch1-3) in control and flow blocked embryos (block with 2.5 uM Nifedipine + 2X Tricaine from 34-38 or 38-42 hpf). Average value of 3 experiments is given in percent.

| **control** | **clutch 1** | **clutch 2** | **clutch 3** | **average in %** |
| --- | --- | --- | --- | --- |
| **34 hpf** | 1 | 2 | 0 | 5.0 |
| **36 hpf** | 6 | 6 | 4 | 6.7 |
| **38 hpf** | 11 | 10 | 5 | 43.3 |
| **40 hpf** | 12 | 12 | 14 | 63.3 |
| **42 hpf** | 15 | 14 | 16 | 75.0 |
| **44 hpf** | 17 | 16 | 17 | 83.3 |
| **46 hpf** | 19 | 20 | 19 | 96.7 |
| **48 hpf** | 19 | 20 | 20 | 98.3 |
|  |  |  |  |  |
| **block 34-38 hpf** | **clutch 1** | **clutch 2** | **clutch 3** | **average in %** |
| **34 hpf** | 1 | 2 | 0 | 5.0 |
| **36 hpf** | 2 | 2 | 0 | 6.7 |
| **38 hpf** | 4 | 3 | 0 | 11.7 |
| **40 hpf** | 9 | 9 | 1 | 31.7 |
| **42 hpf** | 14 | 10 | 10 | 56.7 |
| **44 hpf** | 16 | 16 | 13 | 75.0 |
| **46 hpf** | 19 | 19 | 15 | 88.3 |
| **48 hpf** | 20 | 20 | 18 | 96.7 |
|  |  |  |  |  |
| **block 38-42 hpf** | **clutch 1** | **clutch 2** | **clutch 3** | **average in %** |
| **34 hpf** | 2 | 3 | 0 | 8.3 |
| **36 hpf** | 5 | 6 | 4 | 25.0 |
| **38 hpf** | 8 | 9 | 5 | 36.7 |
| **40 hpf** | 9 | 10 | 6 | 41.7 |
| **42 hpf** | 10 | 11 | 7 | 46.7 |
| **44 hpf** | 10 | 12 | 12 | 56.7 |
| **46 hpf** | 16 | 17 | 19 | 86.7 |
| **48 hpf** | 18 | 20 | 19 | 95.0 |
